# Prognostic and clinical significance of HER-2 low expression in early-stage gastric cancer

**DOI:** 10.1186/s12885-022-10262-7

**Published:** 2022-11-12

**Authors:** Tao Yang, Rui Xu, Junhao You, Fang Li, Bing Yan, Jia-nan Cheng

**Affiliations:** 1 Department of Oncology, Hainan Hospital of Chinese PLA General Hospital, Sanya, 572013 Hainan China; 2 Institute of Cancer, Xinqiao Hospital, Third Military Medical University, Chongqing, 400037 People’s Republic of China

**Keywords:** Gastric cancer, HER-2, Prognosis, Clinical characteristics

## Abstract

**Introduction:**

Gastric cancer is the most fifth common tumor worldwide. Human epidermal growth factor receptor 2 (HER2) overexpression is associated with poor prognosis and clinical characteristics in gastric cancer. Nevertheless, the biology of HER2-low expression has not reported in gastric cancer.

**Materials and methods:**

A total of 157 patients with early-stage gastric cancer were retrospectively analyzed. The associations between HER-2 low expression and clinical characteristics were analyzed by Chi-square test. And the prognostic value of HER-2 low expression and clinical characteristics in disease-free survival (DFS) and overall survival (OS) were analyzed by univariate and multivariate Cox regression analysis.

**Results:**

Of 157 patients with early-stage gastric cancer, 31.8% had HER2-low tumors and 50.3% had HER2-negative tumors. HER2-low expression was associated with age, histological differentiation, tumor location and Ki-67 index. However, HER2-low expression was not associated with DFS or OS in early-stage gastric cancer.

**Conclusion:**

HER2-low expression might result in distinct biology, but it was not an independent prognostic factor of DFS or OS in early-stage gastric cancer.

## Introduction

Gastric cancer is one of the most common malignant tumors of digestive system. The incidence and mortality rate rank the fifth and fourth among all malignancies, respectively [1]. Human epidermal growth factor receptor 2 (HER2) overexpression has been reported in 10–30% of gastric cancer patients [2, 3]. Currently, chemotherapy plus anti-HER2 antibody have been the standard treatment in advanced gastric cancer patients with HER-2 overexpression. The result of ToGA study revealed that trastuzumab plus chemotherapy significantly improved overall survival than chemotherapy alone in patients with HER-2 positive gastric cancer [4]. Moreover, trastuzumab treatment did not result in significantly increase of drug-related cardiac adverse effects [4].

Activation of HER-2 signal pathway might be involved in tumor cell proliferation, differentiation, and vascular and lymphatic angiogenesis [5]. Previous studies reported that HER-2 overexpression correlated with tumor location, histologic subtype and tumor grade in gastric cancer [3, 6]. However, the prognostic role of HER-2 in gastric cancer still remains controversial due to conflicting results in several studies [6–8].

Recently, trastuzumab deruxtecan (T-DXd; DS-8201), as a novel anti-HER2 antibody drug conjugates (ADCs) have demonstrated a superior efficacy in breast cancer patients with HER-2 low expression [9]. The encouraging results expand the targetability of HER-2 to a much wider population. In addition, T-DXd treatment could result in significant improvements in objective response rate (ORR) and overall survival (OS) in patients with HER2-positive gastric cancer [10]. Although about 10% patients receiving T-DXd treatment had drug-related interstitial lung disease or pneumonitis, most events were grade 1–2 and tolerant [10]. Moreover, T-DXd has also shown some clinical activity in patients with HER2-low gastric cancer in the DESTINY-Gastric01 trial.

Nevertheless, the biology of HER2-low expression has not reported in gastric cancer. In this study, we retrospectively analyzed the correlation of HER-2 low expression and clinical characteristics, and its prognostic significance in early-stage gastric cancer.

## Patients and methods

### Patients enrollment and data collection

Between January 2012 and December 2020, a total of 157 patients with early-stage gastric cancer (stage I-III) were investigated in Hainan Hospital of PLA General Hospital. All of patients had received curative surgery and adjuvant chemotherapy of oxaliplatin and 5-fluorouracil, except for those were clarified as T1N0M0. Clinical characteristics were collected, including age, gender, differentiation degree, TNM stage, HER-2 expression and Ki-67 index. This study was supervised by the ethics committee of Hainan Hospital of PLA General Hospital, and written informed consent was not needed since this was a retrospective study.

### Detection of HER-2 expression

In this study, HER-2 expression was determined by immunohistochemistry (IHC) in 142 of 157 surgical specimens. Additionally, HER-2 expression was examined in at least 3 slides by two independent pathologists to minimize the error of intratumorally heterogeneity. HER-2 immunostaining was scored as 0, 1 + , 2 + and 3 + by the universal scoring system as previously reported [11]. Moreover, tumor tissues with scores of 2 + were further detected by fluorescence in situ hybridization (FISH). Scores of 0 were considered negative for HER-2expression, score of 1 + and 2 + (FISH-) were considered low expression, and scores of 3 + and 2 + (FISH +) were considered positive.

### Follow-up of disease-free survival and overall survival

Patients were evaluated during follow-up by computed tomography (CT) of lung, abdomen and pelvic cavity. Disease-free survival (DFS) was defined as the time from the beginning of operation until the appearance of recurrence. Overall survival was defined as the time from the beginning of operation until death. The last follow-up time was on August 31th, 2022, and the medium follow-up time was 62.6 months (range: 11.7–130.4 months).

### Statistical analysis

The associations between HER-2 expression and clinical characteristics were analyzed by Chi-square test and Fisher exact test if the theoretical number is less than 5. Kaplan–Meier survival curves were used to estimate DFS and OS, and univariate and multivariate Cox regression analysis were used to determine the associations between clinicopathological and survival. All the statistical analyses were conducted by using SPSS 20.0 software.

## Results

### Clinicopathological characteristics in overall population

A total of 157 patients with early gastric cancer were enrolled, and their baseline characteristics were shown in Table 1. There were 91 (58.0%) older patients (Age ≥ 60), and the average age was 61 (28–88) years. And most of patients were male (73.9%), with lymph node metastasis (73.9%), poor differentiation (63.1) and stage III (55.4%) in this study. In the overall population, 13 patients (8.3%) had HER2-positive tumors, 50 patients (31.8%) had HER2-low tumors, and 79 patients (50.3%) had HER2-negative tumors, while HER-2 expression of 15 patients (9.6%) was unknown.

**Table 1:** Clinical characteristics of patients with early-stage gastric cancer

Characteristics	n (%)
**Total**	157 (100)
**Age**	
≥ 60y	91 (58.0)
< 60y	66 (42.0)
**Sex**	
Male	116 (73.9)
Female	41 (26.1)
**Tumor location**	
Cardia	33 (21.0)
Corpus	58 (37.0)
Antrum	66 (42.0)
**Differentiation**	
Poor	99 (63.1)
Well	58 (36.9)
**Tumor invasion**	
T1	18 (11.5)
T2	20 (12.7)
T3	73 (46.5)
T4	46 (29.3)
**Lymph node**	
N0	41 (26.1)
N +	116 (73.9)
**TNM stage**	
I	27 (17.2)
II	43 (27.4)
III	87 (55.4)
**HER-2 expression**	
Negative	79 (50.3)
Low	50 (31.8)
Positive	13 (8.3)
Unknown	15 (9.6)

### Clinicopathological characteristics in HER2-low vs HER2-0 tumors

The associations of HER2-low expression and clinicopathological characteristics were summarized in Table 2. HER2-low tumors were significant more common in older patients compared to HER-2 negative tumors (70% vs 49.3%, *P* = 0.021). Moreover, there were more well-differentiated tumors in patients with HER-2 low expression than those without HER-2 expression (44% vs 25.3%, *P* = 0.027). In addition, HER2-low tumors had higher Ki-67 index compared to HER2-0 tumors (70% vs 49.3%, *P* = 0.021). Conversely, less HER-2 low tumors were located in antrum than HER-2 negative tumors (34% vs 49.4%, *P* = 0.032). However, no significant difference was observed between the 2 groups in terms of sex, tumor invasion, lymph node metastasis and TNM stage.

**Table 2:** Clinical characteristics of patients with HER2-low vs HER2-0 expression

	HER2-0	HER2-low	*P* value
**Age**			**0.021**
≥ 60y	39	35	
< 60y	40	15	
**Sex**			0.154
Male	56	41	
Female	23	9	
**Tumor location**			**0.032**
Cardia	13	11	
Corpus	20	29	
Antrum	17	39	
**Differentiation**			**0.027**
Poor	59	28	
Well	20	22	
**Tumor invasion**			0.304
T1	11	3	
T2	12	8	
T3	36	20	
T4	20	19	
**Lymph node**			0.668
N0	20	11	
N +	59	39	
**TNM stage**			0.15
I	18	5	
II	17	15	
III	44	30	
**Ki-67 index**			**0.021**
Low	40	15	
High	39	35	

### Disease outcome of HER2-low tumors vs HER2-0 tumors

Then the disease-free survival (DFS) and overall survival (OS) analysis were performed in these 129 patients. In univariate analysis of DFS, histological differentiation (HR: 1.93[1.08–3.45], *P* = 0.044), tumor invasion depth (HR: 0.15[0.08–0.28], *P* = 0.001), lymph node metastasis (HR: 0.08[0.04–0.14], *P* < 0.001) and TNM stage (HR: 0.21[0.12–0.37], *P* < 0.001) were all associated with poor survival. Further multivariate Cox analysis revealed that lymph node metastasis (HR: 0.08[0.04–0.14], *P* < 0.001) and TNM stage (HR: 0.21[0.12–0.37], *P* < 0.001) were independently associated with poor survival. However, HER-2 low expression was not associated with DFS in early-stage gastric cancer (HR: 0.90[0.47–1.70], *P* = 0.741) (Table 3).

**Table 3:** Univariate and multivariate disease-free survival analysis

	Univariate			Multivariate		
	HR	95%CI	P	HR	95%CI	P
Age(≥ 60 vs < 60)	0.85	0.47–1.51	0.484			
Sex (M vs F)	1.35	0.72–2.52	0.377			
Differentiation (Poor vs Well)	1.93	1.08–3.45	**0.044**			
Tumor invasion (T1-2 vs T3-4)	0.15	0.08–0.28	**0.001**	4.20	0.98–18.00	0.053
Lymph node (N0 vs N +)	0.08	0.04–0.14	** < 0.001**	3.56	1.87–4.25	**0.038**
TNM stage (I-II vs III)	0.21	0.12–0.37	** < 0.001**	4.88	2.03–11.71	**0.000**
Ki-67 index (Low vs High)	0.76	0.40–1.45	0.402			
Her-2 (0 vs Low)	0.90	0.47–1.70	0.741			

Similarly, the univariate analysis of OS revealed that poor differentiation (HR: 1.31[1.16–3.60], *P* = 0.009), deep tumor invasion (HR: 0.13[0.06–0.25], *P* < 0.001), lymph node metastasis (HR: 0.07[0.04–0.14], *P* < 0.001) and stage III (HR: 0.24[0.13–0.44], *P* < 0.001) were also associated with poor survival in early-stage gastric cancer. Final multivariate analysis identified lymph node metastasis as bearing prognostic importance (HR: 13.76[1.88–100.5], *P* = 0.01). However, HER-2 low expression was not associated with OS in early-stage gastric cancer (HR: 1.11[0.60–2.06], *P* = 0.73) (Table 4).

**Table 4:** Univariate and multivariate overall survival analysis

	Univariate			Multivariate		
	HR	95%CI	P	HR	95%CI	P
Age(≥ 60 vs < 60)	0.68	0.36–1.29	0.226			
Sex (M vs F)	1.13	0.59–2.22	0.719			
Differentiation (Poor vs Well)	1.31	1.16–3.60	**0.009**			
Tumor invasion (T1-2 vs T3-4)	0.13	0.06–0.25	** < 0.001**			
Lymph node (N0 vs N +)	0.07	0.04–0.14	** < 0.001**	13.76	1.88–100.5	**0.010**
TNM stage (I-II vs III)	0.24	0.13–0.44	** < 0.001**			
Ki-67 index (Low vs High)	0.82	0.42–1.60	0.55			
Her-2 (0 vs Low)	1.11	0.60–2.06	0.73			

## Discussion

HER-2 is a transmembrane protein with tyrosine kinase activity to mediate cell growth and differentiation and encoded by *ERBB-2* (Erb-B2 receptor tyrosine kinase 2) gene. HER-2 overexpression or *ERBB-2* amplification is universal in many cancers, including breast cancer [12], colorectal cancer [13], lung cancer [14], ovarian cancer [15], gastric or gastroesophageal junction cancer [16], and so on. Moreover, HER-2 overexpression has been reported to be associated with poor prognosis in patients with breast cancer [17], prostate cancer [18], and ovarian cancer [19]. Although several studies reported the prognostic significance of HER-2 overexpression in gastric cancer [6], other studies reported converse conclusions [8, 20].

As approvement of anti-HER2 ADCs drugs in treatment of breast cancer patients, the traditional negative expression of HER-2 has been classified as two subgroups, HER2-0 for tumors scored IHC 0 and HER2-low for tumors scored IHC 1 + or 2 + with a nonamplified FISH assay. Recently, a large cohort study analyzed the biology significance of HER2-low expression in early-stage of breast cancer [21]. Although the prognostic value was not observed, tumors with HER2-low expression exhibited many different clinical characteristics compared to those with HER2-0 expression, including sex, ER expression, histology type, tumor grade and germline mutation. However, the biology significance of HER2-low expression in gastric cancer remains unclear.

To our knowledge, this study firstly reported the clinical and prognostic significance of HER-2 low expression in gastric cancer. We found that HER-2 low expression was associated with age, hitological differentiation, Ki-67 index and tumor location, but not with sex, tumor invasion, lymph node metastasis or TNM stage. The association of HER2-low expression with tumor differentiation is consistent with that of HER2-overexpression in previous studies [7, 22]. Previous studies report that HER2-overepxression tumors are more common in gastroesophageal junction (GEJ) than HER2-negative tumors [3]. In our study, the vulnerable sites of HER2-low tumors are cardia and corpus, while HER2-0 tumors are antrum. And the higher Ki-67 index might be contributed to the function of HER-2 in promoting tumor cell proliferation [23].

Nevertheless, HER-2 low expression was not an independent factor of DFS and OS in early-stage gastric cancer, which is consistent with the findings in breast cancer [21]. The results might be explained in many respects. Firstly, the HER2-0 tumors include those faintly expressing HER-2 in 10% or less of tumor cells according to the latest guidelines [24], which might also activate downstream signal to promote tumor progression. Secondly, distinguishing HER2-0 (IHC0) and HER2-low (especially IHC1) might be difficult and not inaccurate [25], which might result in the incorrect subgroup. Thirdly, the intratumoral heterogeneity is commonly seen in gastric cancer [26]. Although testing in at least 3–4 slides was recommended to minimize the error caused by intratumorally heterogeneity [27], more exhaustive and accurate determination of HER-2 expression remains an open problem.

Unlike in breast cancer, gastric cancer patients with HER-2 overexpression might not benefit from majority of anti-HER2 agents other than trastuzumab [4]. Pertuzumab combined with trastuzumab and chemotherapy did not prolong PFS or OS in first-line treatment for metastatic gastric cancer [28]. Similarly, lapatinib combined with chemotherapy did not improve survival outcomes in first-line or second-line treatment in gastric cancer [29, 30]. Moreover, continued treatment of trastuzumab cloud not prolong survival in gastric cancer patients with disease progression [31]. The heterogeneous of HER2 expression might be the critical factor that limit the efficacy of HER2-targeted treatments in gastric cancer patients [32, 33].

As the first ADC targeting HER-2, trastuzumab emtansine (T-DM1) resulted in effective clinical responses in breast cancer patients with HER-2 overexpression [34], but not in gastric cancer patients [35]. However, as a novel ADC durg, T-DXd treatment results in significant improvements in ORR and OS in patients with HER2-positive gastric cancer [10]. Moreover, T-DXd has also shown some clinical activity in patients with HER2-low gastric cancer. Therefore, there is clinical importance to distinguish HER-2 low tumors from HER-2 negative tumors in gastric cancer patients. Nevertheless, more patients and studies are still needed to verify our conclusion.

## Conclusion

HER2-low expression might result in distinct biology, but it was not an independent prognostic factor of DFS or OS in early-stage gastric cancer.

## Data Availability

The datasets supporting the conclusions of this article are included within the article.

## Abbreviations

HER-2: Human epidermal growth factor receptor 2
ADCs: Antibody drug conjugates
ORR: Objective response rate
OS: Overall survival
DFS: Disease-free survival
IHC: Immunohistochemistry
FISH: Fluorescence in situ hybridization
ERBB-2: Erb-B2 receptor tyrosine kinase 2
